# Decrease in the Antioxidant Capacity in Beverages Containing Tea Extracts during Storage

**DOI:** 10.1100/2012/361698

**Published:** 2012-09-10

**Authors:** Tomas Nekvapil, Vladimir Kopriva, Vladimir Boudny, Martin Hostovsky, Petr Dvorak, Ladislav Malota

**Affiliations:** Department of Biochemistry, Chemistry and Biophysics, Faculty of Veterinary Hygiene and Ecology, University of Veterinary and Pharmaceutical Sciences Brno, Palackeho 1–3, 612 42 Brno, Czech Republic

## Abstract

The aim of this work was to determine antioxidant capacity of beverages containing black, white, and green tea extracts using the photochemiluminescence method, and to monitor its changes based on the storage temperature and time. Samples were stored at two different temperatures (refrigerated at 4°C and laboratory temperature 22°C), analyzed after opening of the original package, and consequently after 4 and 7 days. Results of the antioxidant capacity are expressed as the standard equivalents, that is, ascorbic acid in mmol/L. The highest mean value of the antioxidant capacity was found after opening of the original package in fruit-juice-enriched samples and totaled 9.793 mmol/L. This group revealed significant dependence (*P* < 0.05) not only on the storage time, but also temperature. In samples without added fruit juices containing preservatives the value was 0.428 mmol/L. This group showed significant dependence (*P* < 0.05) on the decrease of antioxidant capacity only when based on the storage time. Samples without fruit juices or preservatives showed significant decrease in the antioxidant capacity (*P* < 0.05) after 4 days of storage based on the storage time. The dependence on temperature was revealed only after 7 days of storage.

## 1. Introduction

 Antioxidant capacity is an overall ability of organisms or food to catch free radicals and prevent their harmful effect. Antioxidative effect includes protection of cells and cellular structures against harmful effect of free radicals, especially oxygen and nitrogen. Substances with antioxidative properties are called antioxidants. They are contained in food and food supplements, most commonly in fruits, vegetables, rice, wine, meat, eggs, and other foodstuff of plant and animal origin.

 Antioxidative systems include antioxidative enzymes, that is, superoxiddismutase (EC 1.15.1.1.), catalase (EC 1.11.1.6.), glutathione peroxidase (EC 1.11.1.9.), glutathione-S-transferase (EC 2.5.1.18.), and nonenzymatic substrates, such as glutathione, uric acid, lipoic acid, bilirubin, coenzyme Q, vitamin C (L-ascorbic acid), vitamin A (retinol), vitamin E (tocopherol), flavonoids, carotenoids (e.g., astaxanthin, zeaxanthin), lycopene, phosvitin, bioflavonoid resveratrol, teine compounds in green tea, and others. Some biomolecules are also considered biologically active and clinically significant antioxidants, for example, transferrin, ferritin, lactoferrin, ceruloplasmin, hemopexin, haptoglobin, and uric acid. Antioxidative, that is, favorable effect of selected antioxidants, was studied in vivo by, for example, Terziev et al. [1], Nguyen-Deroche et al. [2] and Meira de-Faria et al. [3], and in vitro by Gondoin et al. [4].

 The aim of our work was to determine antioxidant capacity of beverages containing tea extracts using the photochemiluminescence method (PCL), and to assess its changes during their storage based on the temperature and storage time. The main antioxidants in beverages containing tea extracts are ascorbic acid and flavonoids. In case of fruit juices, which can be added to tea extracts, they can have natural or synthetic form [5].These components are usually detected as of water-soluble antioxidant fractions [6].

## 2. Materials and Methods 

### 2.1. Materials

 The total of 12 samples of beverages containing black, white, and green tea extracts from closed original PET bottles were analyzed. Each sample was obtained twice, for two different storage temperatures (i.e., refrigerated at 4°C and at the laboratory temperature 22°C), and analyzed at the same time intervals (i.e., after opening of the sealed bottle, and then on the 4th and 7th day). 

*Following are the characteristics of ice tea samples.*

**Group A includes Samples 1–7 Containing Black Tea Extract**

sample A1—black tea with peach, sample A2—black tea with lemon, sample A3—black tea with peach, sample A4—black tea with lemon, sample A5—black tea with peach, sample A6—black tea with mango, and sample A7—black tea with pear.

**Group B includes Samples 1 and 2 Containing White Tea Extract**

sample B1—white tea with pomegranate, sample B2—white tea with apricot.

**Group C includes Samples 1–3 Containing Green Tea Extract**

Sample C1—green tea with citrus, sample C2—green tea with lemon and cactus, sample C3—green tea with aloe vera.

### 2.2. Methods

The analysis was carried out using the analytic system Photochem (Analytik Jena, Germany). It uses the photochemiluminescence (PCL) method, which serves for the determination of water-soluble antioxidant capacity (ACW) and allows quantification of the antioxidant state. Its principle is based on the production of free radicals (superoxid radical) induced by optical excitation of the photosensitive solution. These radicals are partly eliminated by their reaction with antioxidants in the sample. Remaining radicals cause luminescence, which is detected and used to calculate the antioxidant capacity of the sample. The results were compared with the calibration curve, quantified, and expressed in equivalents of standard, that is, ascorbic acid for ACW antioxidants. ACW antioxidants were determined using the ACW Kit supplied by the Jena AG Company. The kit contained the following:

- reagent R1—diluent (sample solvent)—stored at 4°C;
- reagent R2—reaction buffer—stored at 4°C;
- reagent R3—photosensitizer to be dissolved in 750 *μ*L reagent R1 (sufficient for approximately 40 measurements)—stored at −20°C and after diluting at 4°C;
- reagent R4—standard—ascorbic acid (vitamin C) stored at −20°C.

The PCL method was used for the determination of ascorbic acid standards, and with the use of linear regression, calibration curve was constructed. Statistical evaluation was performed using Microsoft Excel 2010 (Microsoft Corporation, Inc., Mountain View, CA, USA). The significance of the relationships between the storage time and temperature was tested using the analysis of variance (Anova) at the significance level *P* < 0.05.

#### 2.2.1. Preparation of Samples

The amount of 10 *μ*L of sample was mixed with 1500 *μ*L R1 and 1000 *μ*L R2. The reaction mixture was mixed using a vortex mixer, and immediately before its sucking into the analytic system Photochem, 25 *μ*L of reagent R3 were added. Each sample was prepared in three replicates. 

## 3. Results and Discussion

Antioxidants and antioxidant capacity of food have become important especially in connection with civilization illnesses [7]. Many authors therefore focus on the determination of antioxidant capacity [8–11].

 The PCL method was first described by Popov and Lewin [12]. Benzie et al. [8] and Langley-Evans [13], and Robinson et al. [14] studied the antioxidant capacity of dark and green tea according to the flavonoids content. Vertuani et al. [6] obtained values ranging from 4.27 to 30.2 mmol/L (ascorbic acid equivalent) in the studied tea extracts. The lowest value was measured in Oolong, Chinese brown tea, and the highest in Bancha, Japanese green tea. 

**Figure 1:**
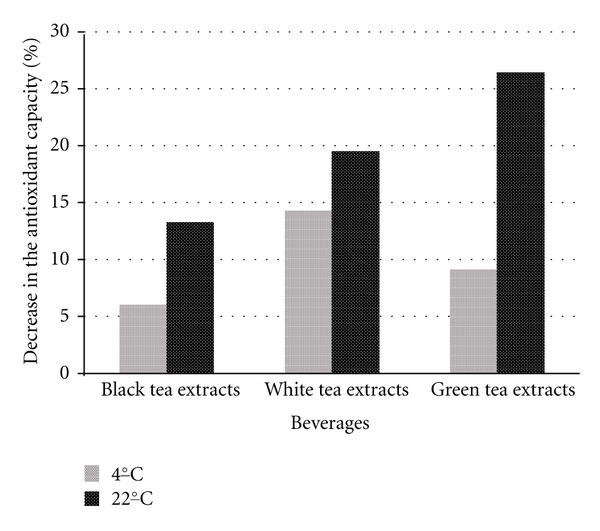
Decrease in the antioxidant capacity in the stored beverages in %, day 0–4.

**Figure 2:**
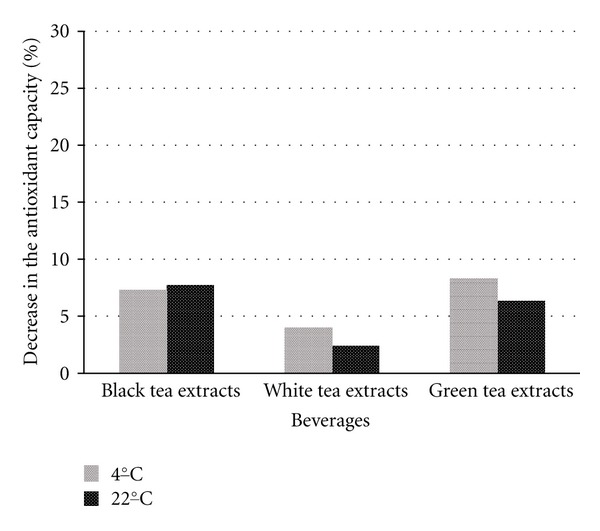
Decrease in the antioxidant capacity in the stored beverages in %, day 4–7.

Manufacturers of our analyzed samples stated that samples must be refrigerated and consumed within 3 days. Therefore we conducted the measurement immediately after opening of original packages and consequently on the fourth and for the control seventh day of the experiment. Results of the antioxidant capacity are expressed in the ascorbic acid equivalents (mmol/L) and stated in Table 1. Decrease in the antioxidant capacity is expressed in percentage and shown in Figures 1 and 2. 

The highest value of antioxidant capacity was observed at the onset of the experiment in samples containing fruit juices, without preservatives (i.e., samples A7, B1, B2, C1, C2, and C3). This juice is probably a natural source of substances with antioxidative properties, as confirmed by Graham [5]. When compared with the mean initial antioxidant capacity of these samples (9.924 mmol/L), samples without added fruit juices containing preservatives (i.e., samples A3, A4, A5, and A6) showed significantly lower values (*P* < 0.05), that is, 0.428 mmol/L, and samples without added fruit juices or preservatives (i.e., samples A1 and A2) the value showed 0.646 mmol/L.

Samples containing fruit juices revealed a decrease in their antioxidant capacity (*P* < 0.05) due to the storage time already after 4 days (11.5%, when stored at 4°C; and by 23.7%, when stored at 22°C). This group showed significant dependence (*P* < 0.05) on temperature as well. The total decrease (*P* < 0.05) after 7 days of storage was 17.1% (4°C) and 28.3% (22°C), respectively.

Samples, which did not contain fruit juices but contained preservatives (samples A3, A4, A5, and A6), showed an insignificant decrease in the antioxidant capacity depending on the storage temperature. On the other hand, there was a significant decrease (*P* < 0.05) of the antioxidant capacity depending on the storage time. 

Samples A1 and A2, which did not contain both fruit juices and preservatives, revealed significant decrease (*P* < 0.05) in their antioxidant capacity when stored at laboratory temperature after 4 days (12.2%). There was a significant decrease (*P* < 0.05) in their antioxidant capacity after 7 days of storage, depending on the storage temperature (9.9%, when stored at 4°C; and by 19.6%, when stored at 22°C).

The antioxidative capacity decreased most significantly in beverage containing green and white tea extracts during the first 4 days of storage (Figure 1). The antioxidant capacity of beverage containing black tea extracts decreased after another 3 days of storage more than in white and green extracts (see Figure 2). The highest decrease was observed in the refrigerated sample B2, that is, by 23% after 4 days of storage and by 27.1% after 7 days of storage. In the samples stored at laboratory temperature, the highest decrease was observed in the sample C2, by 40.8% after 4 days and by 43.1% after 7 days of storage.

Figures 1 and 2 show that the greatest decrease of the antioxidant capacity occurred in samples with green and white tea extracts during the first 4 days of the storage, at both temperatures. The following 3 days of storage revealed smaller changes of the antioxidant capacity in these samples, which can be attributed to the decrease of substances with antioxidant activity during the first days of storage. The decrease of antioxidant capacity and therefore substances with antioxidant capacity corresponds with the study by del Caro et al. [15], who monitored changes in the content of flavonoids, vitamin C, and antioxidant capacity during the storage of samples in a fridge. 

Coyle et al. [16], Almajano et al. [17], Narotzki et al. [18], and Espinosa et al. [19] confirmed in their studies the positive impact of white and green teas on the antioxidant activity and also on many biological as well as biochemical processes in the organism. The content of phenolic substances also depends on the method of their extraction from tea leaves, which was confirmed by Rusak et al. [20]. 

## 4. Conclusions

Results imply the positive impact of adding fruit juices on the antioxidant capacity of beverages containing white, black, and green tea extracts. The decrease in antioxidant capacity was more significant in samples stored at laboratory temperature, especially during the first 4 days of their storage. After 3 more days of storage, there was a significant decrease in antioxidant capacity only in samples containing black tea extracts. Samples containing preservatives showed statistically significant difference only in the storage time. 

The importance of antioxidant capacity of food, including beverages, is very topical due to the growing understanding of the role of antioxidants in the prevention of noninfectious civilization diseases. 

## Figures and Tables

**Table 1:** Results of ACW determination in individual groups of samples. Mean values are expressed as equivalents of ascorbic acid (mmol/L) when stored at the temperature *T* = 4°C and *T* = 22°C, and storage time *t* = 0, 4, and 7 days, respectively.

Sample	Storage time (days)			Temperature (°C)
	0	4	7
A1	0.250 ± 0.010	0.240 ± 0.006	0.232 ± 0.004^Δ^	4
		0.228 ± 0.002*	0.201 ± 0.002^∗Δ^	22
A2	1.042 ± 0.027	0.950 ± 0.042	0.912 ± 0.018^∗Δ^	4
		0.880 ± 0.029*	0.840 ± 0.020^∗Δ^	22
A3	0.016 ± 0.001	0.015 ± 0.003	0.014 ± 0.002	4
		0.013 ± 0.002	0.012 ± 0.001*	22
A4	0.575 ± 0.014	0.528 ± 0.016*	0.480 ± 0.009*	4
		0.498 ± 0.011*	0.474 ± 0.012*	22
A5	0.522 ± 0.001	0.520 ± 0.003	0.492 ± 0.004*	4
		0.508 ± 0.005*	0.486 ± 0.009*	22
A6	0.598 ± 0.019	0.582 ± 0.002	0.440 ± 0.021*	4
		0.534 ± 0.022*	0.410 ± 0.029*	22
A7	8.496 ± 0.171	7.355 ± 0.029^∗Δ^	7.330 ± 0.037^∗Δ^	4
		6.435 ± 0.041^∗Δ^	6.115 ± 0.069^∗Δ^	22
B1	8.915 ± 0.075	8.420 ± 0.059^∗Δ^	8.080 ± 0.032^∗Δ^	4
		7.890 ± 0.036^∗Δ^	7.690 ± 0.016^∗Δ^	22
B2	15.650 ± 0.057	12.050 ± 0.041^∗Δ^	11.400 ± 0.016^∗Δ^	4
		11.350 ± 0.113^∗Δ^	10.950 ± 0.036^∗Δ^	22
C1	9.087 ± 0.108	8.864 ± 0.091^∗Δ^	8.310 ± 0.029^∗Δ^	4
		7.115 ± 0.051^∗Δ^	6.280 ± 0.054^∗Δ^	22
C2	11.230 ± 0.041	9.030 ± 0.022^∗Δ^	8.640 ± 0.036^∗Δ^	4
		6.650 ± 0.04^∗Δ^	6.400 ± 0.029^∗Δ^	22
C3	5.380 ± 0.075	5.100 ± 0.090^∗Δ^	4.280 ± 0.054^∗Δ^	4
		4.480 ± 0.075^∗Δ^	4.070 ± 0.033^∗Δ^	22

The values are means of 3 measurements ± S.D. (standard deviation).

*Statistically significant dependence on the storage time.

^Δ^Statistically significant differences between storage temperatures.
